# Trends in behaviour change techniques for implementing and de-implementing healthcare practices using audit and feedback

**DOI:** 10.1186/s43058-026-01059-x

**Published:** 2026-07-28

**Authors:** Hamish Duncan, Andrea M. Patey, Jacob Crawshaw, Justin Presseau, Jeremy M. Grimshaw, Noah Ivers, Fabiana Lorencatto, Vivi Antonopoulou

**Affiliations:** 1 Division of Psychology and Language Sciences in Faculty of Brain Sciences, University College London, 26 Bedford Way, London, WC1H 0AP UK; 2 Department of Medicine, Quality and Safety, IWK Health Centre, Halifax, NS Canada; 3 Methodological and Implementation Research Program, Ottawa Hospital Research Institute, Ottawa, ON Canada; 4 Department of Medicine, University of Ottawa, Ottawa, ON Canada; 5 Department of Family and Community Medicine, Women’s College Hospital, Toronto, Canada; 6 NIHR Policy Research Unit (PRU) in Behavioural and Social Sciences, Centre for Behaviour Change (CBC), Department of Clinical, Educational and Health Psychology, University College London, 1-19 Torrington Place, London, WC1E 7HB UK

**Keywords:** Audit and feedback, Implementation, De-implementation, Healthcare professionals, Quality improvement, Intervention design, Behaviour change

## Abstract

**Background:**

Audit and feedback (A&F) is a widely used quality improvement strategy in which a summary of healthcare professionals’ (HCPs) practice is provided to support behaviour change. However, its application and effectiveness within randomized controlled trials (RCTs) of practice-change interventions vary considerably. Examining variation in the behaviour change techniques (BCTs) used within A&F interventions and the contexts in which they are applied may help optimise their design and delivery. Therefore, this study explored associations between the BCTs used in A&F interventions, the direction of behaviour change (implementation versus de-implementation), and target behaviour type.

**Methods:**

An exploratory secondary analysis was conducted using data from 261 RCTs of A&F interventions, originally extracted as part of a Cochrane systematic review. Binary logistic regression models investigated whether different BCTs were more frequent in interventions for the implementation versus de-implementation of HCP practice. These analyses were repeated in subgroups of different behaviour types (e.g. prescribing, testing/examinations).

**Results:**

Descriptive analyses of 261 RCTs (942 target behaviours) indicated that BCTs were used with broadly similar frequency for implementation (*n* = 732 behaviours) and de-implementation (*n* = 179 behaviours). Due to substantial clustering within studies, regression model estimates were unstable and not interpreted inferentially. Across the full sample, most BCTs showed minimal differences in use between implementation and de-implementation. Subgroup analyses by behaviour type suggested some variation, with certain BCTs (e.g. feedback on outcomes of behaviour) appearing to be used differentially depending on the type of practice being modified. However, overall patterns were largely consistent across behaviour categories.

**Conclusions:**

These findings suggest that A&F interventions typically use similar BCTs regardless of whether they aim to increase or decrease HCP behaviours, and largely irrespective of behaviour type. This may indicate that current A&F intervention design does not systematically account for the direction or nature of behaviour change. While some variation was observed by behaviour type, the absence of clear differentiation highlights a potential mismatch between theoretical expectations and practice. Future research should use theory-informed designs to determine whether tailoring BCTs to behaviour change direction or type improves A&F intervention effectiveness.

## Contributions to the literature

- This study is the first to examine behaviour change techniques (BCTs) use exclusively in audit and feedback (A&F) interventions analysing at the level of individual target behaviours (rather than whole interventions), and accounting for both behaviour change direction, i.e. implementation versus de-implementation, and behaviour type simultaneously. Findings demonstrate that the same BCTs are used for implementation and de-implementation, suggesting practice does not reflect the view that distinct BCTs are required for each.
- This work highlights potential limitations in A&F design and calls for future research testing whether aligning BCTs with behaviour change direction improves effectiveness.

## Introduction

Audit and feedback (A&F) is a type of behaviour change intervention in which a summary of healthcare professionals (HCPs) clinical performance is produced over a specified period (audit) and compared to a standard (e.g., clinical practice guidelines or a benchmark) and the results are communicated back to HCPs (feedback) to promote behaviour change [1–4]. It is used in many healthcare systems internationally to influence HCP behaviour, promoting evidence-based practices and discouraging those that are ineffective [5, 6]. By aligning clinical performance with best practice, A&F has the potential to improve patient outcomes [7–9].

Extensive research has examined the effectiveness and underlying mechanisms of A&F in healthcare [1, 2]. Meta-analyses indicate that, on average, A&F leads to small-to-moderate improvements in clinical practice; however, effects are highly variable [10–12].

This variability likely reflects multiple factors, including the context in which A&F is delivered [13], the characteristics of the target population [14], and, critically, the behaviour change techniques (BCTs) used within interventions [1, 2, 12, 13]. BCTs are “observable, replicable and irreducible components of an intervention that are designed to alter or redirect causal processes regulating behaviour” [15, p. 82], such as providing instructions on how to perform the behaviour (e.g. educational booklet, lectures, workshops on diabetes care), restructuring the physical environment or providing rewards [15, 16]. Recent work has highlighted that a wide range of BCTs are used in A&F interventions [1, 2, 17, 18]. As the active components of behavioural interventions, variation in BCT use may contribute to differences in A&F effectiveness [2, 19].

Despite their importance, the processes by which BCTs are selected in A&F interventions remain poorly understood [19]. This has been attributed, in part, to the limited use of behaviour change theory in intervention design [19–21]. One possibility is that intervention designers implicitly tailor BCT selection based on characteristics of the target behaviour. Two such characteristics are the intended direction of behaviour change and the type of behaviour being targeted [22, 23].

The direction of behaviour change refers to whether an intervention aims to implement a behaviour (i.e., increase its occurrence) or de-implement it (i.e., reduce its frequency) [24]. This distinction has gained increasing attention in recent years [22, 23, 25], with ongoing debate about whether different strategies are required for implementation versus de-implementation. If so, A&F designers may need to consider behaviour change direction when selecting BCTs [22, 23].

In addition, behaviours of a similar type may share underlying determinants [26], meaning that similar BCTs could be effective across related behaviours [27, 28]. Consequently, BCT selection may also vary according to the type of behaviour targeted. Understanding whether and how these factors influence A&F design is important for identifying current practices and informing future optimisation.

A recent review examined these issues across a range of behavioural interventions and found that BCT use varied according to both behaviour change direction and behaviour type [22]. However, this work classified interventions based on a single overarching goal, which may not reflect A&F interventions that often target multiple behaviours simultaneously, some of which are implemented while others are de-implemented [1].

This present study examines whether A&F intervention content differs according to the direction of behaviour change and the type of behaviour targeted. Specifically, we assess, at the level of individual target behaviours, whether: 1) A&F interventions use different BCTs for implementation versus de-implementation; and 2) this relationship varies by behaviour type.

## Methods

### Design

The current study is an exploratory secondary analysis. It utilises a dataset of BCTs used in A&F interventions, produced as part of a recent Cochrane review [1, 2].

### Sample

The original dataset included 287 A&F studies, published before June 2020, which had been selected for an analysis of BCTs in A&F as part of an update of the Cochrane systematic review of A&F interventions [1, 2]. Full details of the search strategy and eligibility criteria are reported elsewhere [12]. The dataset included randomised controlled trials (RCTs) and cluster RCTs in which A&F was used as a standalone or co-intervention to influence HCP behaviour [1, 2].

Of the 287 studies, 261 were included in the present analyses. Data were extracted on target behaviours, intervention content, and study aims. Twenty-six studies were excluded due to incomplete data for these variables, leaving a final sample of 261 studies.

### Measures

#### Intervention content

The dataset describes which of 95 BCTs were used in each intervention. All 93 BCTs in the behaviour change technique taxonomy version 1 (BCTTv1) [17] were used to describe the content of the interventions. Additionally, 2 BCTs (education (unspecified) and feedback (unspecified) were included from an A&F codebook, which was developed specifically to code A&F intervention content [2].

#### Types of target behaviour

The dataset identified all behaviours targeted within each intervention. These were initially grouped into eight categories: prescribing, testing/examination, treatment decision/action, counselling, immunization, referrals, diagnosis, and screening.

The three largest categories (prescribing, testing/examination, and treatment decision/action) were conceptually broad and so were subdivided to enable more granular subgroup analyses. Prescribing was categorised into prophylaxis, immediate treatment, monitoring, and support, reflecting a difference in the immediacy of the action being taken [29]. Testing/examination was divided into examinations, imaging, and laboratory tests. These three subcategories reflect distinctions that were included in many of the papers [30–33] and refer to different categories of techniques used for testing/examination. Finally, treatment decision/action was separated into the categories: medication decisions, surgical decisions, and other. The first two subcategories reflect two large observed groups within the dataset. However, due to the high variability in the treatment decisions/actions category, it was necessary to include an additional category of ‘other’ to describe a large portion of this subgroup. A description of each behavioural category, subcategory, and examples can be found in Table 1.

**Table 1:** Description and examples of the categories and subcategories of target behaviours used within the study

Behaviour subcategory	Description	Example and source
**Prescribing**		
*Prophylaxis*	A prescribing behaviour completed to prevent a disease or future negative outcome	Rates of prophylactic use of oxytocin [34]
*Immediate* *treatment*	A prescribing behaviour completed to have an immediate effect on a patient	Proportion of patient encounters with antibiotic prescribed [35]
*Monitoring*	A prescribing behaviour taken to maintain the quality of longer-term prescriptions	Amiodarone for ≥6 months without a thyroid function test in the past 6 months [36]
*Support*	A behaviour taken to support the patient to adhere to an ongoing treatment	Methotrexate without instructions to take weekly [36]
**Testing/examination**		
*Examination*	A behaviour related to physically examining a patient	Average proportion of paediatric admissions with documentation of weight [37]
*Imaging*	A behaviour related to the use of imaging to examine a patient	% Transthoracic echocardiography ordered that are rarely appropriate [38]
*Laboratory tests*	A behaviour related to the use of laboratory tests for diagnostic information about a patient	Proportion of angina patients with cholesterol checked [39]
**Treatment decision/action**		
*Medication* *decisions*	Behavioural decisions or actions relating to the prescription of medication	% Patients with sepsis receiving antibiotic therapy within 1 hour [40]
*Surgical decisions*	Behavioural decisions or actions relating to the performance of surgical procedures	% Patients with planned caesarean delivery [41]
*Other*	Treatment decisions/actions that do not fit into the above categories	Proportion of patients with Do Not Resuscitate orders in place at time of death [42]
**Counselling**	The provision of advice or training to a patient	Proportion of Asthmatic patients advised to avoid passive smoking [39]
**Diagnosis**	A clinical diagnosis is made given certain criteria are met or relevant information is available	Proportion of asthma diagnoses based on 1 of 3 criteria [39]
**Immunization**	Behaviours directly related to the administration of immunizations	Mean vaccination rate for influenza at each centre [43]
**Referral**	Behaviours related to or the act of providing a patient with a referral to see another specialist	Proportion of patients with a dietician visit in the last 60 months [44]
**Screening**	The act of pre-emptively assessing patients for otherwise unindicated conditions	Proportion of patients at or above target for HbA1c measurement in previous 12 months [45]

Subcategory classifications were made in the first place by the lead author using the full texts of relevant studies. These allocations were then reviewed without blinding by the corresponding author. Due to limited sample sizes within several subcategories, analyses at this level were not undertaken. However, their production is detailed here to support their use in future research.

#### Categorisation of target behaviours as implemented or de-implemented

The original dataset did not include information on whether behaviours were being implemented or de-implemented, so this was coded for in the current study. A binary classification was not always appropriate, as some entries combined multiple behaviours. Where behaviours were affected in the same direction, classification was straightforward. Where behaviours included both implementation and de-implementation (e.g., composite guideline adherence outcomes), these were classified as “mixed” and excluded from analysis. Classification was conducted through review of titles and abstracts, with full texts consulted where necessary.

### Data analysis

#### Research question 1: Are different BCTs used for implementation versus de-implementation?

The primary research question explored whether the same BCTs were used in A&F interventions aimed at de-implementation as those aimed at implementation. To investigate this a series of binary logistic regressions were conducted, wherein whether a target behaviour was being implemented or de-implemented predicted whether a given BCT was applied to that target behaviour. This approach was applied to all BCTs that were used on over 100 target behaviours, as with fewer than 100 uses there was a high chance that a BCT was either always or never used for either implementation or de-implementation, which would have meant that the regression could not accurately estimate coefficients. If, despite this measure, such a situation did arise, the regression would not be run for that BCT. As it was possible that multiple target behaviours were present in the same study, meaning the data was nested, a random effect of study was included within the model. The final regression equation was then:

$${\mathrm{BCT}}_{{\mathrm{Absent}} = 0}^{{\mathrm{Present}} = 1} \sim {\mathrm{Target}}\;{\mathrm{Behaviour}}_{{\mathrm{De-imp}} = 0}^{{\mathrm{Imp}} = 1} + (1|{\mathrm{study}})$$

#### Research question 2: Are different BCTs used for implementation versus de-implementation depending on the type of practice?

The above analysis was then repeated in subgroups, each containing one type of target behaviour, to investigate whether different techniques are used for implementation and de-implementation depending on the type of target behaviour. The categories in the original dataset were analysed first, followed by the subcategories that were generated in this study.

#### Correction for multiple testing

Between the main and subgroup analyses 64 unique regression models were run, which increased the chances of a false positive. As a result, a Bonferroni correction was applied, with alpha being adjusted to 0.0078 [46, 47].

## Results

### Coding of target behaviours

There was a total of 942 target behaviours within the dataset. The intended direction of behaviour change of 749 behaviours was categorised using the stated aims and title of the paper. The remaining 193 were categorised via full-text screening. Following classification, most target behaviours were coded as being implemented (*n* = 732), with fewer de-implemented (*n* = 179), and a small number classified as mixed (*n* = 31). Descriptive statistics for the sample are presented in Table 2.

**Table 2:** Descriptive statistics of the sample resulting from the categorisations made in this study

Target behaviours		Implemented target behaviours	Studies implementing at least one target behaviour		De-implemented target behaviours			Studies de-implementing at least one target behaviour
Prescribing		189	89		115			51
Testing/Examination		216	75		41			24
Treatment Decision/Action		96	48		18			12
Counselling		96	46		2			2
Diagnosis		68	41		1			1
Immunization		45	26		0			0
Referral		13	10		2			1
Screening		9	2		0			0
Whole sample		732	196		179			88

#### Research question 1: Are different BCTs used for implementation versus de-implementation?

The first analysis examined whether the presence of different BCTs predicted if a target behaviour was intended to be implemented or de-implemented. After the removal of “mixed” behaviours, the dataset for this analysis included 911 target behaviours, each coded as implemented (*n* = 732) or de-implemented (*n* = 179). Sixteen BCTs met the inclusion threshold of being used in at least 100 target behaviours.

**Fig. 1:**
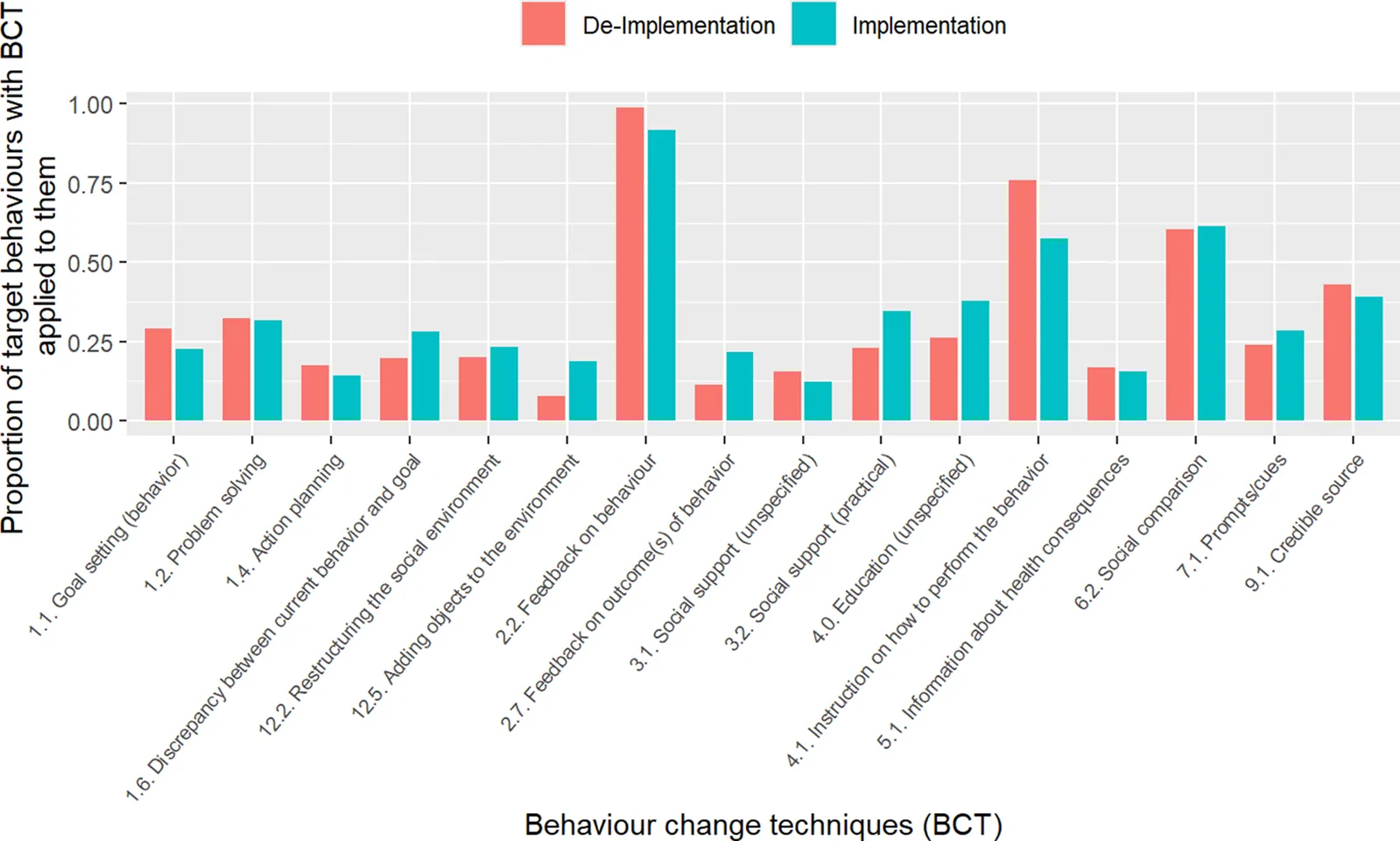
Proportion of total target behaviours and BCT use, by implementation vs. de-implementation. *Note*: The number of target behaviours to which a BCT was applied divided by the total number of target behaviours split by whether the behaviour was being implemented or de-implemented

Across the full sample, BCTs were used with broadly similar frequency for implementation and de-implementation. Differences in the proportion of use between behaviour change directions were generally small across most BCTs (Fig. 1; Table 3).

**Table 3:** Descriptive statistics and outputs from the regression models fit to the whole data sample

BCT	Proportion of implemented behaviours to which BCT was applied	Proportion of de-implemented behaviours to which BCT was applied	Regression coefficient (odds ratio)	95% Confidence interval (odds ratio)	*p*	**σ**_**study**_
1.1 Goal Setting (Behaviour)	22.54%	29.05%	0.76	0.01–46.57	0.894	64.45
1.2 Problem Solving	31.56%	32.40%	1.02	0.03–32.37	0.990	59.21
1.4 Action Planning	14.21%	17.32%	0.80	0.00–243.05	0.940	69.59
1.6 Discrepancy Between Current Behaviour and Goal	28.14%	19.55%	0.82	0.03–23.27	0.908	59.61
2.2 Feedback on Behaviour	91.67%	98.88%	0.36	0.00–15,265.69	0.850	71.91
2.7 Feedback on Outcome of Behaviour	21.72%	11.17%	1.15	0.02–72.15	0.947	62.28
3.1 Social Support (Unspecified)	12.16%	15.64%	0.50	0.00–53.00	0.773	64.86
3.1 Social Support (Practical)	34.42%	22.91%	1.45	0.04–58.09	0.845	60.31
4.1 Instruction on how to Perform a Behaviour	57.65%	75.98%	1.00	0.07–15.16	0.998	53.33
4.0 Education (Unspecified)	37.70%	26.26%	0.95	0.05–16.92	0.971	55.05
5.1 Information about Health Consequences	15.57%	16.76%	0.45	0.01–16.37	0.665	59.45
6.2 Social Comparison	61.34%	60.34%	1.88	0.16–22.70	0.621	51.51
7.1 Prompts and Cues	28.28%	24.02%	0.97	0.03–34.42	0.988	59.99
9.1 Credible Source	39.07%	43.03%	0.73	0.05–10.12	0.813	53.70
12.2 Restructuring the Social Environment	23.09%	20.11%	1.03	0.02–44.41	0.986	60.92
12.5 Adding Objects to the Environment	18.58%	7.82%	1.77	0.01–388.32	0.836	64.58

Across the analyses, the random effect used to control for the nested nature of the data was very large. This likely impacted the estimation of the standard errors of the coefficients, meaning the confidence intervals and *p* values associated with them are unreliable. As a result, interpretation of the results will focus primarily on the coefficient itself and the raw differences in proportion of use.

#### Research question 2: Are different BCTs used for implementation versus de-implementation depending on the type of practice?

**Fig. 2:**
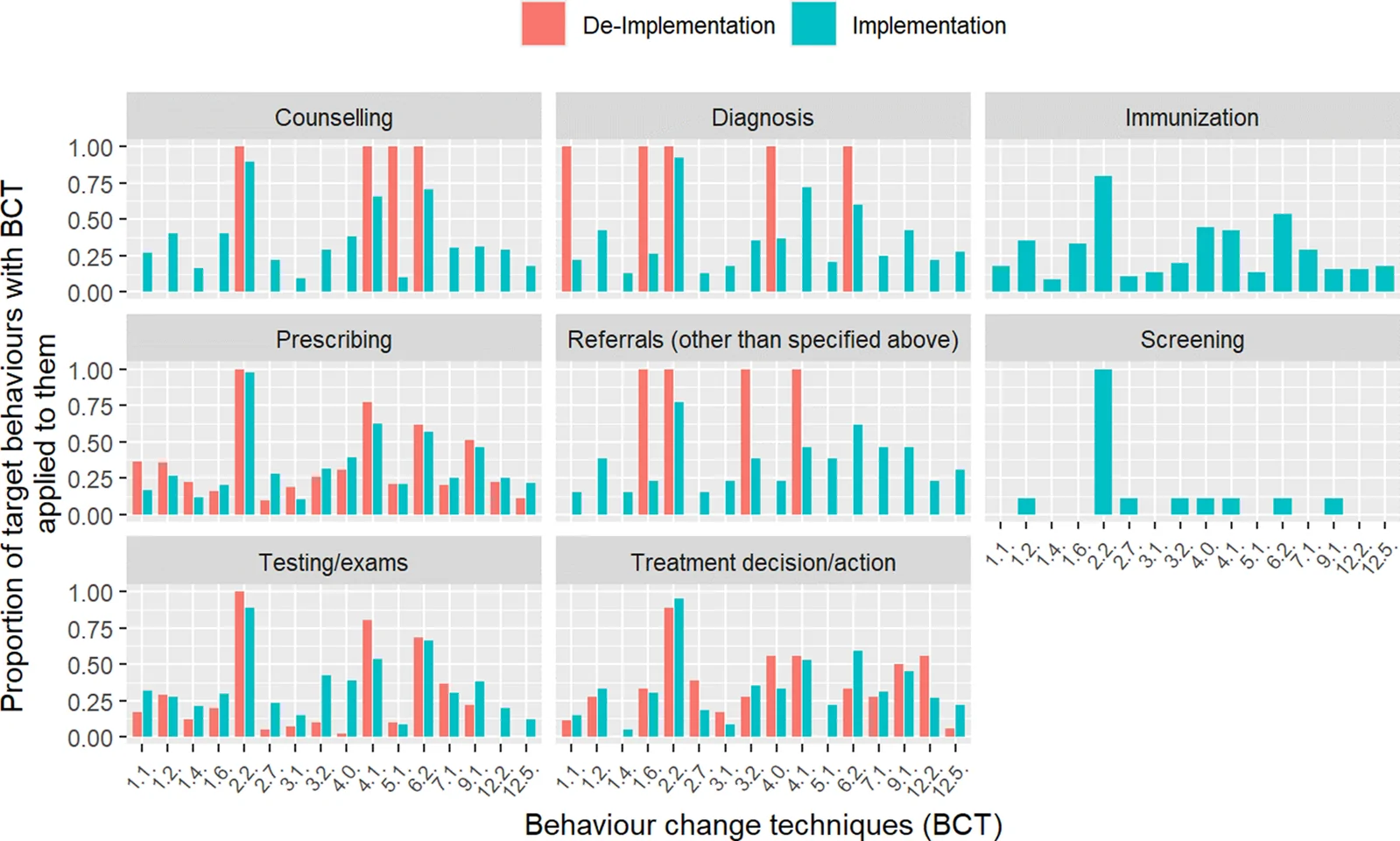
Proportion of different types of behaviour and BCT use by implementation or de-implementation. *Note*. The number of target behaviours to which a BCT was applied divided by the total number of target behaviours split by whether the behaviour was being implemented or de-implemented and the type of behaviour. The BCTs in this graph are depicted by their numerical labels. For a translation of these numbers see Fig. 1

Subgroup analyses were conducted for the three largest behaviour categories: prescribing, testing/examination, and treatment decision/action. Across these categories, patterns of BCT use remained broadly consistent with those observed in the full sample (Fig. 2).

The output of the regressions conducted for the prescribing category can be seen in Table 4. It was not possible to run a regression to assess whether “feedback on behaviour” was used more for implementation or de-implementation, as it was always used for de-implementation. All other planned regressions were conducted.

**Table 4:** Descriptive statistics and outputs from the regression models fit to “prescribing” target behaviours

BCT	Proportion of implemented behaviours to which BCT was applied	Proportion of de-implemented behaviours to which BCT was applied	Regression coefficient (odds ratio)	95% Confidence interval (odds ratio)	*p*	**σ**_**study**_
1.1 Goal Setting (Behaviour)	16.93%	36.52%	0.66	0.00–93.64	0.869	59.89
1.2 Problem Solving	26.46%	35.65%	1.27	0.01–118.90	0.918	55.17
1.4 Action Planning	11.64%	22.61%	1.45	0.00–3,074.60	0.925	66.34
1.6 Discrepancy Between Current Behaviour and Goal	20.11%	15.65%	1.62	0.01–192.02	0.843	55.19
2.2 Feedback on Behaviour	97.88%	100%	-	-	-	-
2.7 Feedback on Outcome of Behaviour	28.04%	9.57%	3.20	0.01–766.52	0.677	55.70
3.1 Social Support (Unspecified)	10.05%	19.13%	0.55	0.00–242.86	0.848	60.74
3.1 Social Support (Practical)	31.22%	26.09%	2.44	0.02–310	0.718	55.10
4.1 Instruction on how to Perform a Behaviour	62.43%	77.39%	0.69	0.02–28.24	0.843	51.57
4.0 Education (Unspecified)	39.15%	30.43%	1.53	0.04–54.07	0.815	48.73
5.1 Information about Health Consequences	20.63%	20.87%	0.76	0.01–62.25	0.901	55.43
6.2 Social Comparison	56.61%	61.74%	0.71	0.03–19.23	0.840	47.60
7.1 Prompts and Cues	24.87%	20.00%	1.60	0.01–188.78	0.847	55.42
9.1 Credible Source	46.56%	51.30%	1.02	0.05–21.52	0.991	45.72
12.2 Restructuring the Social Environment	24.87%	22.61%	1.34	0.01–140.79	0.903	56.08
12.5 Adding Objects to the Environment	21.69%	11.30%	2.00	0.01–654.81	0.814	58.67

In the testing/examinations category, regression analyses could not be conducted for the BCTs “feedback on behaviour”, “restructuring the social environment”, and “adding objects to the environment” due to their either total or lack of use for de-implementation. Additionally, regression models for the BCTs “social comparison” and “credible source” failed to successfully converge. The remaining regression outputs can be seen in Table 5.

**Table 5:** Descriptive statistics and outputs from the regression models fit to “testing and examination” target behaviours

BCT	Proportion of implemented behaviours to which BCT was applied	Proportion of de-implemented behaviours to which BCT was applied	Regression coefficient (odds ratio)	95% Confidence interval (odds ratio)	*p*	**σ**_**study**_
1.1 Goal Setting (Behaviour)	31.48%	17.07%	1.24	0.00–2432.31	0.955	59.40
1.2 Problem Solving	29.27%	27.31%	0.61	0.00–164.43	0.865	54.17
1.4 Action Planning	21.30%	12.20%	0.68	0.00–2,091.67	0.924	63.27
1.6 Discrepancy Between Current Behaviour and Goal	29.63%	19.51%	0.61	0.00–117.25	0.852	53.53
2.2 Feedback on Behaviour	88.43%	100%	-	-	-	-
2.7 Feedback on Outcome of Behaviour	23.15%	4.88%	2.41	0.00–15,297.85	0.844	56.48
3.1 Social Support (Unspecified)	14.81%	7.32%	0.66	0.00–1,828.80	0.918	61.03
3.2 Social Support (Practical)	42.59%	9.76%	2.92	0.00–3,710.25	0.769	53.63
4.1 Instruction on how to Perform a Behaviour	53.24%	80.49%	0.72	0.01–78.25	0.890	47.86
4.0 Education (Unspecified)	38.89%	2.44%	12.13	0.00–1,298,756.08	0.673	50.54
5.1 Information about Health Consequences	8.80%	9.76%	0.44	0.00–500.13	0.820	57.09
6.2 Social Comparison	66.20%	68.28%	-	-	-	-
7.1 Prompts and Cues	30.09%	36.59%	0.85	0.00–274.11	0.955	53.66
9.1 Credible Source	37.96%	21.95%	-	-	-	-
12.2 Restructuring the Social Environment	19.91%	0%	-	-	-	-
12.5 Adding Objects to the Environment	12.04%	0%	-	-	-	-

Finally, in the treatment decision/action category, regression analysis could not be conducted for the BCT “information about health consequences” because it was not used for de-implementation. Further to this, the models for the BCTs “goal setting (behaviour)”, “action planning”, “information about health consequences”, and “social comparison” did not converge. The outputs for the remaining regressions can be seen in Table 6.

**Table 6:** Descriptive statistics and outputs from the regression models fit to “treatment decision/action” target behaviours

BCT	Proportion of implemented behaviours to which BCT was applied	Proportion of de-implemented behaviours to which BCT was applied	Regression coefficient (odds ratio)	95% Confidence interval (odds ratio)	*p*	**σ**_**study**_
1.1 Goal Setting (Behaviour)	14.58%	11.11%	-	-	-	-
1.2 Problem Solving	33.33%	27.78%	1.20	0.00–736.94	0.956	47.69
1.4 Action Planning	5.21%	0%	-	-	-	-
1.6 Discrepancy Between Current Behaviour and Goal	30.21%	33.33%	1.17	0.00–5,600.98	0.971	53.22
2.2 Feedback on Behaviour	94.80%	88.89%	2.32	0.00–554,756.00	0.894	61.15
2.7 Feedback on Outcome of Behaviour	18.75%	38.89%	0.42	0.00–359.96	0.799	51.10
3.1 Social Support (Unspecified)	8.33%	16.67%	0.49	0.00–6,040.11	0.883	55.42
3.2 Social Support (Practical)	35.42%	27.78%	1.42	0.00–911.84	0.916	47.30
4.1 Instruction on how to Perform a Behaviour	53.13%	55.56%	0.83	0.00–333.54	0.951	45.13
4.0 Education (Unspecified)	33.33%	55.56%	0.00	0.00–0.00	<0.001*	46.07
5.1 Information about Health Consequences	21.88%	0%	-	-	-	-
6.2 Social Comparison	59.38%	33.33%	-	-	-	-
7.1 Prompts and Cues	31.25%	27.78%	1.72	0.00–6,340.68	0.897	49.99
9.1 Credible Source	44.79%	50.00%	0.54	0.00–161.54	0.830	46.64
12.2 Restructuring the Social Environment	27.08%	55.56%	-	-	-	-
12.5 Adding Objects to the Environment	21.88%	5.56%	4.11	0.00–2,347,828.98	0.834	55.02

## Discussion

The first aim of this study was to investigate whether the same BCTs are used within A&F interventions in healthcare when implementing versus de-implementing target behaviours. While regression analyses were conducted, instability in model estimates limited their interpretability. Accordingly, findings are interpreted descriptively.

Results consistently indicated that most BCTs were used with similar frequency for implementation and de-implementation, with generally small differences observed across techniques. This suggests that, in current practice, A&F interventions do not systematically differentiate BCT selection according to the direction of behaviour change.

The absence of consistent differentiation in BCT use is itself an important finding. Several explanations may account for this pattern. First, it may reflect that implementation and de-implementation rely on broadly similar behavioural mechanisms, meaning that similar BCTs are appropriate for both increasing and decreasing behaviours [28, 48], as implied in most classic theories of behaviour change [23]. If so, the distinction between implementation and de-implementation may be less meaningful from a behaviour change perspective than is often assumed [25, 49, 50]. However, this interpretation should be treated with caution. The present study classified implementation and de-implementation at the level of individual target behaviours, whereas previous work (e.g., [22]), classified interventions at the level of the whole study. This methodological difference may account for some differences in findings between studies, and limits the extent to which conclusions can be directly compared across them.

Second, it is possible that the BCT taxonomy alone does not fully capture the features of A&F interventions that differentiate strategies for increasing versus decreasing behaviours [51, 52]. A&F interventions are complex and often involve characteristics such as feedback format, dose/intensity, comparator selection, and delivery context, which may not be adequately represented through BCT classification alone [2].

Third, context is closely related to this limitation of the BCT taxonomy: many of the features that differentiate A&F interventions, such as who delivers feedback, the frequency and mode of delivery, and the organisational setting, operate at a contextual level that sits outside the BCT framework. Thus, variation in A&F intervention design may be better explained by contextual factors, than by the direction of behaviour change per se. Context encompasses a wide range of features including organisational culture, resource availability, professional norms, baseline performance levels, and broader healthcare system characteristics, all of which are known to moderate A&F effectiveness [53], obscuring any effect that the direction of behaviour change has. If interventions operating in systematically different contexts tend to adopt different BCTs, aggregating across those contexts, as this study necessarily does, may obscure meaningful variation. Furthermore, contextual factors may determine not only which BCTs are selected but how they are implemented in practice—aspects that BCT coding alone cannot capture. Moreover, such factors are often poorly reported in published studies, limiting the ability to examine their role [54].

However, inspection of the results (see Fig. 1 and Table 3), suggests that the majority of the BCTs included in the analyses were used with similar frequency for implementation and de-implementation, with differences generally below 10%. This is broadly consistent with previous research, which suggests that most BCTs are used with similar frequency for implementation and de-implementation [22]. However, some discrepancies do exist. In Patey et al.’s study [22], “feedback on behaviour” and “restructuring the social environment” were found to be associated with implementation and de-implementation respectively. In contrast, in this study, differences of only ~3% and ~7% were observed with both cases showing the reverse directionality. These minor discrepancies may be attributable to methodological differences (e.g., inclusion criteria, analytic approach) or to the present study’s focus solely on A&F interventions. Overall, the results of this study are consistent with the notion that, in current practice, the same BCTs are applied regardless of the intended direction of behaviour change.

The second aim was to examine whether patterns of BCT use differed across categories of target behaviour. Analyses suggested that BCTs were used with similar frequency for implementation and de-implementation across behaviour types, with no consistent pattern of differentiation.

Some variation in BCT use was observed within behaviour-specific subgroups. However, these differences were inconsistent and should be interpreted cautiously given smaller sample sizes and instability in model estimates. While certain BCTs appeared to vary across contexts, these patterns were not robust and may reflect random variation rather than meaningful differences.

As compared to the whole sample, there are several larger discrepancies in BCTs being used for either implementing or de-implementing. For example, “social support (practical)” is used approximately 30% more for implementing than de-implementing in the testing and examinations category. However, due to the smaller sample sizes in this analysis, it might be anticipated that there would be bigger differences between use for implementation and de-implementation due to chance. This makes interpretation of the proportion of target behaviours to which a given BCT was applied difficult. Even accounting for the greater variability expected with smaller subgroup samples, the majority of BCTs continued to show similar rates of use for implementation and de-implementation across behaviour types, suggesting this pattern is not contained to the full-sample analysis. One interesting pattern can be observed in the data for the BCT “feedback on outcome of behaviour”, although this should be interpreted with caution due to this papers lack of reliable inferential statistics. Its use appears to differ across behaviour-specific subgroups, with slightly greater use for implementation in the testing/examinations and prescribing categories, but greater use for de-implementation in the treatment decisions/actions category. This may suggest some context-specific variation in how feedback on behavioural outcomes is applied but given the small subgroup sizes and instability in model estimates, this pattern may also reflect random variation rather than a meaningful difference. As this study did not assess BCT effectiveness, further interpretation is beyond the scope of the analysis.

Overall, the findings of this study indicate that current A&F practice rarely employs different BCTs according to the intended direction of behaviour change. Furthermore, this pattern is relatively stable across the different types of practice that A&F interventions are used on. One exception to this may be “feedback on outcome of behaviour”. However, without the use of inferential statistics this cannot be assessed.

### Strengths and limitations

A key strength of this study is its use of a large, systematically coded dataset of target behaviours from A&F interventions in healthcare, enabling detailed analyses at the level of individual behaviours. This granularity allowed us to avoid the simplifying assumption that all behaviours within an A&F intervention share the same change direction and to model behaviour-level predictors while accounting for study-level clustering.

However, several limitations should be acknowledged. First, the granularity of our approach also served to limit the study, as we had to include random effects in our analyses. These random effects were large and explained the majority of variance in our dependent variables. They also impacted the estimation of standard errors in the regression analyses. As a result, it was not possible to use inferential statistics to determine if there were any true effects within the data.

Our analyses were also limited by only including BCTs which were used 100 or more times. While this threshold was important to ensure sufficient statistical power, it also meant that only the most frequently used BCTs were considered. While this threshold was important to ensure sufficient data for modelling, it may have excluded BCTs that were used frequently in one direction but rarely or never in the other, precisely the pattern that would indicate meaningful differentiation between implementation and de-implementation. For example, a BCT used 90 times for implementation and not at all for de-implementation would have been excluded despite representing a potentially important difference. This means the analyses may have missed some of the most informative variation in BCT use, and future research should consider alternative inclusion criteria that are sensitive to asymmetric patterns of use across behaviour change directions.

There is no theoretical rationale to suggest that these BCTs should be more suited for either implementation or de-implementation than others within the taxonomy. Furthermore, it is plausible that frequently used BCTs are incorporated almost by default into A&F interventions, whereas those that are less commonly included may be used more intentionally with greater consideration of A&F intervention characteristics such as the intended direction of behaviour change.

Finally, it is also important to note that the study was not designed to determine whether particular BCTs are used more successfully for implementing versus de-implementing target behaviours. Ideally, it would be possible to not only understand what current practice in A&F intervention design was, but also what best practice would be. However, this study serves only to do the former.

### Implications for future research and practice

This study offers partial evidence that the same BCTs are used for implementing and de-implementing target behaviours regardless of their types within A&F interventions. This is an important finding with potential implications for both theory and practice. If certain BCTs would be more suitable for implementing target behaviours than de-implementing [22, 23, 26, 27], then current practices could be improved by tailoring A&F intervention strategies accordingly. Understanding which BCTs work best in each context could enhance the effectiveness of behaviour change interventions. Conversely, if BCTs are equally suitable for implementing and de-implementing, as reflected in current practice, then this finding has theoretical significance. It would suggest that BCTs operate through mechanisms that are broadly applicable across both processes, challenging assumptions that different strategies are needed for implementation versus de-implementation. The current study did not test which of these two hypotheses is accurate, so future research should aim to investigate this premise.

Future research should examine whether tailoring BCT selection to behaviour change direction, behaviour type, or contextual factors improves A&F intervention effectiveness. To do so meaningfully, studies will need to operationalise context systematically, for example by stratifying analyses by healthcare setting, and capture the organisational and professional conditions in which A&F is delivered. Additionally, examining combinations of BCTs, rather than individual techniques, may provide further insight into how A&F interventions are designed and function in practice [55, 56].

## Conclusion

The present findings suggest that A&F interventions do not typically tailor BCT selection according to the intended direction of behaviour change or the type of behaviour targeted. This consistency in practice may indicate that A&F intervention design does not currently reflect theoretical distinctions between implementation and de-implementation. Whether this represents an appropriate generalisable approach or a missed opportunity to optimise A&F intervention effectiveness remains unclear. Future research should determine whether aligning BCT selection with behaviour change direction and context improves outcomes, thereby informing more targeted and theoretically grounded A&F intervention design.

## Data Availability

The datasets used and/or analysed during the current study are available from the corresponding author on reasonable request.

## Abbreviations

A&F: Audit and Feedback
BCT(s): Behaviour Change Technique(s)
HCP(s): Health Care Professional(s)
BCTTV1: Behaviour Change Technique Taxonomy Version 1
VIF: Variance Inflation Factor
